# Evolutionary scenarios for the specific recognition of nonhomologous endogenous peptides by G protein–coupled receptor paralogs

**DOI:** 10.1016/j.jbc.2024.108125

**Published:** 2024-12-25

**Authors:** Akira Shiraishi, Azumi Wada, Honoo Satake

**Affiliations:** Bioorganic Research Institute, Suntory Foundation for Life Sciences, Kyoto, Japan

**Keywords:** machine learning, G protein–coupled receptor, peptide hormone, MAS-related GPCR, molecular evolution

## Abstract

Several peptides interact with phylogenetically unrelated G protein–coupled receptors (GPCRs); similarly, orthologous GPCRs interact with distinct ligands. The neuropeptide substance P (SP) activates both NK1R and another unrelated primate-specific GPCR, MRGPRX2. Furthermore, MRGPRX1, a paralog of MRGPRX2, recognizes BAM8–22 (bovine adrenal medulla peptide 8–22), which has no evolutionary relatedness to SP. To elucidate the molecular basis and evolutionary history of this phylogenetically unrelated ligand selectivity, we developed a systematic procedure, the “interaction determinant likelihood score” system, which estimates the amino acid residues responsible for peptide–GPCR interactions predicted by peptide descriptor–incorporated support vector machine, our original machine learning–based peptide–GPCR interaction predictor. An interaction determinant likelihood score–based approach followed by pharmacological validation revealed the determinant residues for the ligand selectivity of SP-MRGPRX2 (F3.24 and G4.61) and BAM8–22–MRGPRX1 (L1.35). Molecular phylogenetic analysis revealed that the MRGPRX1 of common ancestral primates recognized BAM8–22, whereas the ancestral Cercopithecinae MRGPRX1 lost its interaction with BAM8–22 because of the loss of L1.35. The SP–MRGPRX2 interaction emerged in the common ancestors of Euarchonta, and, thereafter, the interaction of MRGPRX2 with both SP and BAM8–22 was acquired *via* substitution with L1.35 in several lineages. Collectively, the present study unraveled the molecular mechanisms and evolution of ligand specificity in evolutionary unrelated GPCRs.

Specific interactions between peptidic ligands (neuropeptides and peptide hormones) and the cognate receptors are primary steps of various biological events in the nervous, neuroendocrine, and endocrine systems in metazoans. Most peptide receptors belong to the G protein–coupled receptor (GPCR) superfamily (1, 2). The diversification and gain of peptides and GPCRs in each species are believed to be involved in common and species-specific signaling (3, 4). Most paralogous GPCRs share high sequence similarity, and their cognate peptide ligands also conserve consensus sequence motifs (5, 6). However, several evolutionary unrelated peptides interact with each GPCR paralog. Likewise, several evolutionarily distant GPCRs share a peptide ligand, which also expands the diversity of peptide–GPCR interaction networks (4, 7, 8).

The protooncogene MAS-related GPCR family includes typical GPCRs with species-specific distributions and unique nonparalogous interactions. It comprises four (MRGPRA–C and MRGPRH) rodent-specific subfamilies, four (MRGPRD–G) mammal-specific subfamilies, and one (MRGPRX) primate-specific subfamily (7, 8). In primates, human MRGPRX1 mediates the itch sensation induced by drugs (9) and chronic pain inhibition in the dorsal root ganglia (10), and MRGPRX2 transmits itchy stimuli through mast cell degranulation (11, 12) and neuroinflammatory pain (13). Furthermore, MRGPRX2 is distributed in primates, including Hominidae, Cercopithecinae, and Platyrrhini (14). In contrast, MRGPRX1 is present in Hominidae and Cercopithecidae (15), but not in Platyrrhini, which diverged before the divergence of Hominidae and Cercopithecidae (16).

MRGPRX1 and MRGPRX2 specifically interact with evolutionarily unrelated peptides: bovine adrenal medulla peptide 8–22 (BAM8–22) and substance P (SP), respectively (12, 17, 18). Notably, MRGPRX1, despite 65% sequence identity with MRGPRX2, does not interact with SP. Instead, SP interacts with another GPCR, NK1R (19) (Fig. S1), which is evolutionarily distant from the MRGPRX family (13). These findings indicated that several peptide–MRGPRX interactions cannot be predicted by sequence similarity or the molecular phylogenetic relationships of ligands or GPCRs. Consequently, the exploration of the molecular basis of the distinct ligand selectivity of MRGPRXs will provide crucial clues to the biological significance and evolutionary scenario of these primate-specific GPCRs. To date, three-dimensional structure analyses have revealed molecular mechanisms of GPCR activation *via* conserved residues (20, 21). However, limited information of crystal structures of GPCRs hinders the elucidation of the molecular basis of marked differences in the ligand preferences of orthologous GPCRs, including MRGPRXs, using three-dimensional structures. Recent deep learning–based AlphaFold analysis cannot also be universally employed because of the difficulty of predicting the structure of residues with post-translational modification and the small amount of information on GPCR structures (22).

Recently, we developed a machine learning system for peptide–GPCR interactions, peptide descriptor (PD)–incorporated support vector machine (SVM), and identified 11 novel species-specific neuropeptide–GPCR pairs by a combination of PD-incorporated SVM prediction and experimental validation (23, 24). Since PD-incorporated SVM predicted the interactions accurately only with amino acid sequences for peptides and GPCRs, it can recognize amino acids involved in peptide–GPCR interactions (23, 24). In this study, we predicted amino acid residues involved in the respective peptide–GPCR interactions using a novel method, "interaction determinant likelihood (IDL) score," which was developed from PD-incorporated SVM, and validated these predictions by cell-based signaling assays for "determinant candidate-exchanged (DCE)" mutants of MRGPRX1 and MRGPRX2. Ultimately, we elucidated the amino acids that are crucial for the recognition of BAM8–22 and SP by MRGPRX1 and MRGPRX2 and the evolutionary process of the acquisition of the ligand specificity by these two GPCRs.

## Results

### Strategy for interaction determinant identification using IDL score and DCE mutation

**Figure 1:**
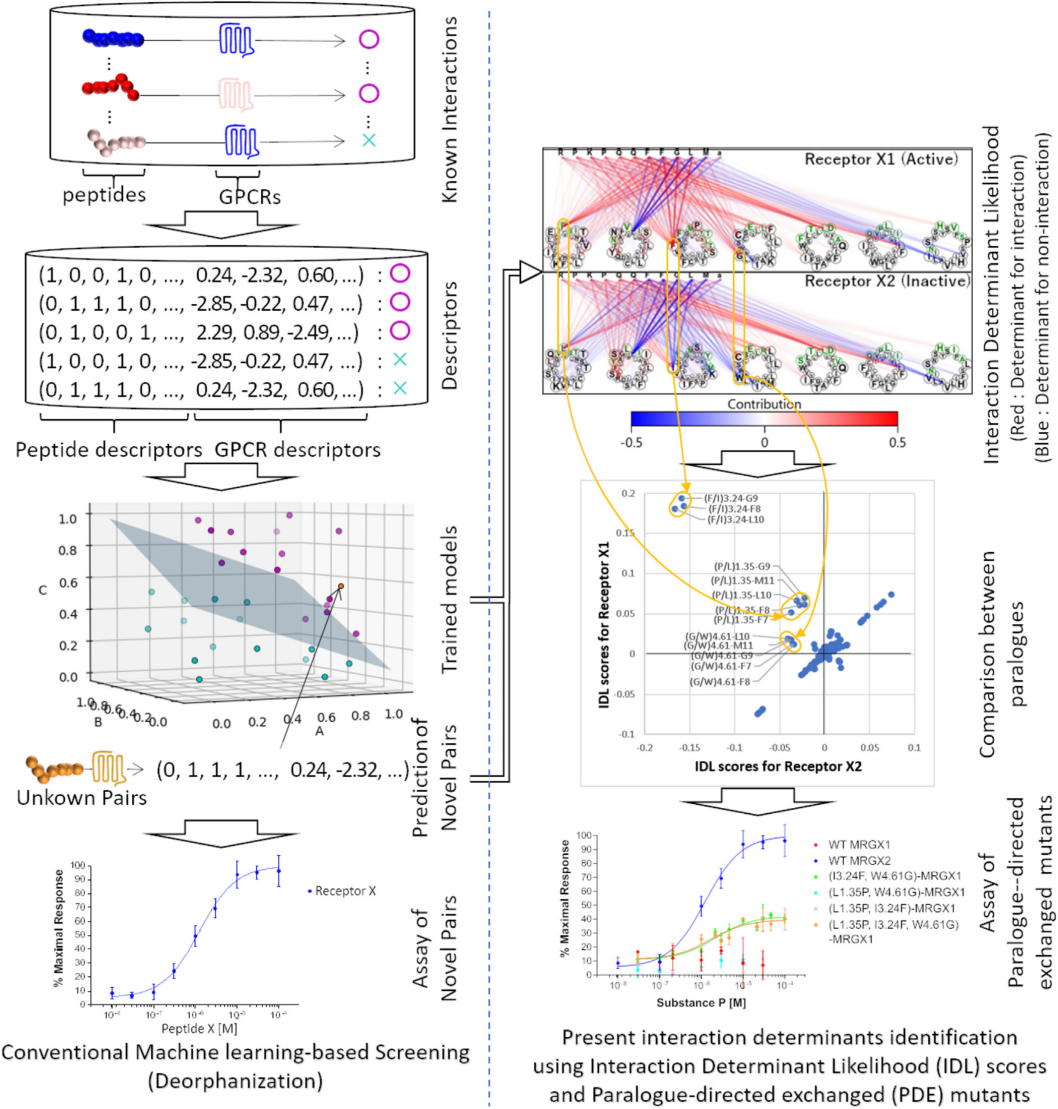
**The strategy for identifying interaction determinants.** In conventional machine learning–based screening, novel interactions are predicted using prediction models trained with descriptor-converted known interactions and then experimentally assayed. In the present strategy, each peptide–GPCR residue pair for the peptide–GPCR interaction of interest is given an interaction determinant likelihood (IDL) score. The residue pairs with positive IDL scores are interpreted as the residues responsible for the interaction. Then, the differentially scored residues between paralogs are assessed by the experimental validation of determinant candidate-exchanged (DCE) mutants. GPCR, G protein–coupled receptor.

In canonical machine learning, novel interactions are predicted using models trained on descriptor-converted known interactions. However, the mechanisms underlying these predicted interactions remain unknown. We previously developed the PD-incorporated SVM to predict peptide–GPCR interactions (23). In this system, the peptide and GPCR sequences are converted into our originally developed descriptors. Each element of the descriptors can be mapped to the corresponding residue position in the peptide and GPCR sequences (23). Then, the GPCRs and peptides converted into descriptors are used as inputs to learn the peptide–GPCR interactions using linear SVM. Linear SVM can extract the weight of each element of the input descriptors for prediction. Therefore, we defined the weight of each residue pair in the predictions of the PD-incorporated SVM as the "IDL score" (Figs. 1 and S2). Briefly, positive and negative IDL scores indicate positive and negative contributions of each residue in pairs between a peptide and a GPCR to the peptide–GPCR interaction prediction, respectively.

To evaluate the correlation of IDL scores with peptide–GPCR interaction, we compared the IDL scores for the interaction between neurotensin and its cognate receptor, NTR1, using the cocrystallized structures (25). The cocrystallized structures demonstrated that Y146^3.29^ and R327^6.54^ directly interacted with neurotensin (25), and R311^6.38^ participated in the interaction indirectly by forming part of the ligand cavity (26). These three residues were among the top five NTR1 residues with the highest IDL scores (R311^6.38^, G144^3.27^, R327^6.54^, W130^2.67^, and Y146^3.29^) for the interaction (Fig. S3), confirming the reliability of the IDL score method for predicting amino acids responsible for peptide–GPCR interactions. This result indicated that the residues predicted to be responsible for interaction act as interaction determinants. For further evaluation, we compared the GPCR residues with high IDL scores to the experimentally identified GPCR mutation effects on peptide recognition in GPCRdb. The performance of the IDL score method for predicting interaction-decreasing mutations was evaluated using the area under the receiver operating characteristic curve (AUC), resulting in an AUC of 0.881 (Fig. S4). This AUC evaluation included predictions for distantly related GPCRs in the phylogenetic tree, such as MC4R, CCKBR, GNRHR, C5AR, and V2R. The high AUC demonstrated the versatility of the IDL score method in predicting amino acids responsible for interactions between a wide variety of GPCRs and their cognate peptide ligands. We also predicted the pathogenicity of these mutations using Alphamissense, which produced an AUC of 0.71, lower than that of our proposed IDL scores (Fig. S4). Collectively, these results corroborated that the IDL score method accurately predicts amino acids involved in peptide–GPCR interactions across diverse peptides and GPCRs. Thus, we used IDL-score methods to predict the residues responsible for the specificity of peptide–MRGPRX interactions.

### IDL score–based prediction of amino acid residues that are essential for interaction with SP in MRGPRX2

**Figure 2:**
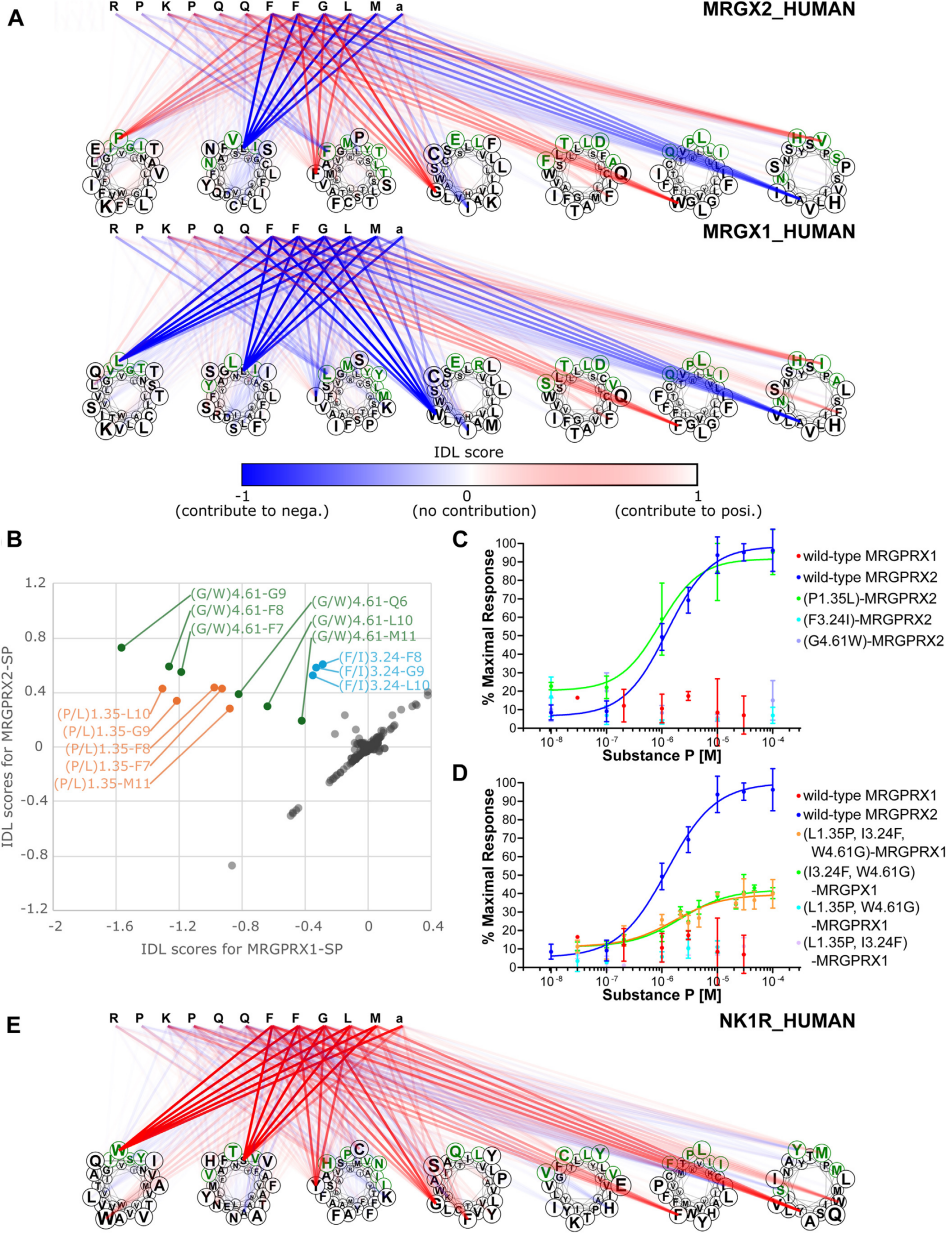
**Estimated IDL scores and DCE validation for the MRGPRX1–SP pair and the MRGPRX2–SP pair.***A*, IDL scores of the MRGPRX2–SP pair and MRGPRX1–SP pair. Transmembrane residues are plotted with *circles*, and cavity-exposed residues are highlighted in *green*. Contribution scores for each peptide and GPCR residue pair are displayed in the heatmap. *B*, scatter plot of IDL scores for the MRGPRX2–SP pair and the MRGPRX1–SP pair. MRGPRX2-specific high-scoring residues are plotted in *orange*, *blue*, and *green* based on receptor residue position. *C*, dose-dependent responses of MRGPRX2 mutants to substance P. *D*, dose-dependent responses of MRGPRX1 mutants to substance P. *E*, IDL scores of the NK1R–SP pair. DCE, determinant candidate-exchanged; IDL, interaction determinant likelihood; SP, substance P.

The human MRGPRX family of GPCRs consists of MRGPRX1–4 (7, 8), but only MRGPRX1 and MRGPRX2 are known to be activated by specific peptides: BAM8–22 (17) and SP (18), respectively. To elucidate the molecular mechanisms underlying this ligand selectivity, we estimated the IDL scores of MRGPRX family GPCRs for interaction with SP and BAM8–22. We initially compared the IDL scores of the SP–MRGPRX2 and SP–MRGPRX1 pairs, an active and an inactive compound–protein interaction (CPI) pair, respectively (Fig. 2*A*).

The comparison of IDL scores between SP–MRGPRX1 and SP–MRGPRX2 displayed residues P1.35, F3.24, and G4.61 of MRGPRX2 as the high-scoring residues interacting with Q6 to M11 of SP (Fig. 2*B*, *upper left area*). In contrast, the corresponding residues of MRGPRX1 (L1.35, I3.24, and W4.61) showed negative values with Q6 to L10 of SP (Fig. 2, *A* and *B*). These results suggest that residues P1.35, F3.24, and G4.61 are critical for the interaction of MRGPRX2 with SP. To verify these putative determinants, we assessed the pharmacological features of mutants of MRGPRX1 and MRGPRX2; for example, MRGPRX1 mutants were generated by replacing the predicted SP-binding determinants (P1.35, F3.24, and/or G4.61) in wildtype MRGPRX2 with the corresponding residues from MRGPRX1 and *vice versa* for MRGPRX2 mutants (Fig. 2*C* and Table 1). Such mutants are designated as "DCE" mutants. Their pharmacological properties were assessed based on calcium responses to SP in COS-7 cells expressing MRGPRX1/2 proteins fused to Gαq16 at the C terminus. Thus, the Gαq16-fused MRGPRX1/2 and their DCE mutants are independent of the effects on downstream G protein selectivity.

**Table 1:** EC_50_ and the maximal response of MRGPRX1/2 mutants to SP

GPCR	LogEC50 (EC50)	Maximal response
MRGPRX2 WT	−5.90 ± 0.10 (1.26 μM)	100.00 ± 6.85%
MRGPRX1 WT	ND	ND
(P1.35L)-MRGPRX2	−6.03 ± 0.31 (0.93 μM)	91.75 ± 13.75%
(F3.24I)-MRGPRX2	ND	ND
(G4.61W)-MRGPRX2	ND	ND
(L1.35P, I3.24F, W4.61G)-MRGPRX1	−5.78 ± 0.25 (1.66 μM)	39.53 ± 3.00%
(I3.24F, W4.61G)-MRGPRX1	−5.65 ± 0.19 (2.24 μM)	42.1 ± 2.28%
(L1.35P, W4.61G)-MRGPRX1	ND	ND
(L1.35P, I3.24F)-MRGPRX1	ND	ND

ND, not determined.

Values are expressed as the means ± standard errors of three replicate analyses, and values that were not determined are expressed as ND.

An MRGPRX2 DCE mutant, (P1.35L)-MRGPRX2 mutant, showed equipotent dose responsiveness to wildtype MRGPRX2. Conversely, the (F3.24I)-MRGPRX2 and (G4.61W)-MRGPRX2 DCE mutants showed no response to SP, indicating that F3.24 and G4.61 are crucial for the interaction with SP. Subsequently, we introduced MRGPRX2-type DCE mutations into MRGPRX1 (L1.35P, I3.24F, and/or W4.61G; Fig. 2*D* and Table 1). Intriguingly, the triple mutations in MRGPRX1 resulted in an Emax response to SP of 39.53 ± 3.00% compared with the interaction of intact MRGPRX2 with SP. Moreover, the double DCE mutant (I3.24F, W4.61G)-MRGPRX1 also had an Emax response of 42.1 ± 2.28% to SP, whereas (L1.35P, W4.61G)-MRGPRX1 and (L1.35P, I3.24F)-MRGPRX1 exhibited no response to SP (Fig. 2*D* and Table 1). Collectively, we concluded that F3.24 and G4.61 are critical determinants for the interaction of MRGPRX2 with SP. Furthermore, NK1R, a phylogenetically unrelated SP-selective receptor (27), was shown to have high IDL scores at Y3.24 and G4.61 that were conserved between MRGPRX2 and NK1R (Fig. 2*E*), suggesting that NK1R and MRGPRX2 share a common SP recognition mechanism, regardless of their evolutionary unrelatedness.

### IDL score–based prediction of amino acid residues essential for interaction with BAM8–22 in MRGPRX1

**Figure 3:**
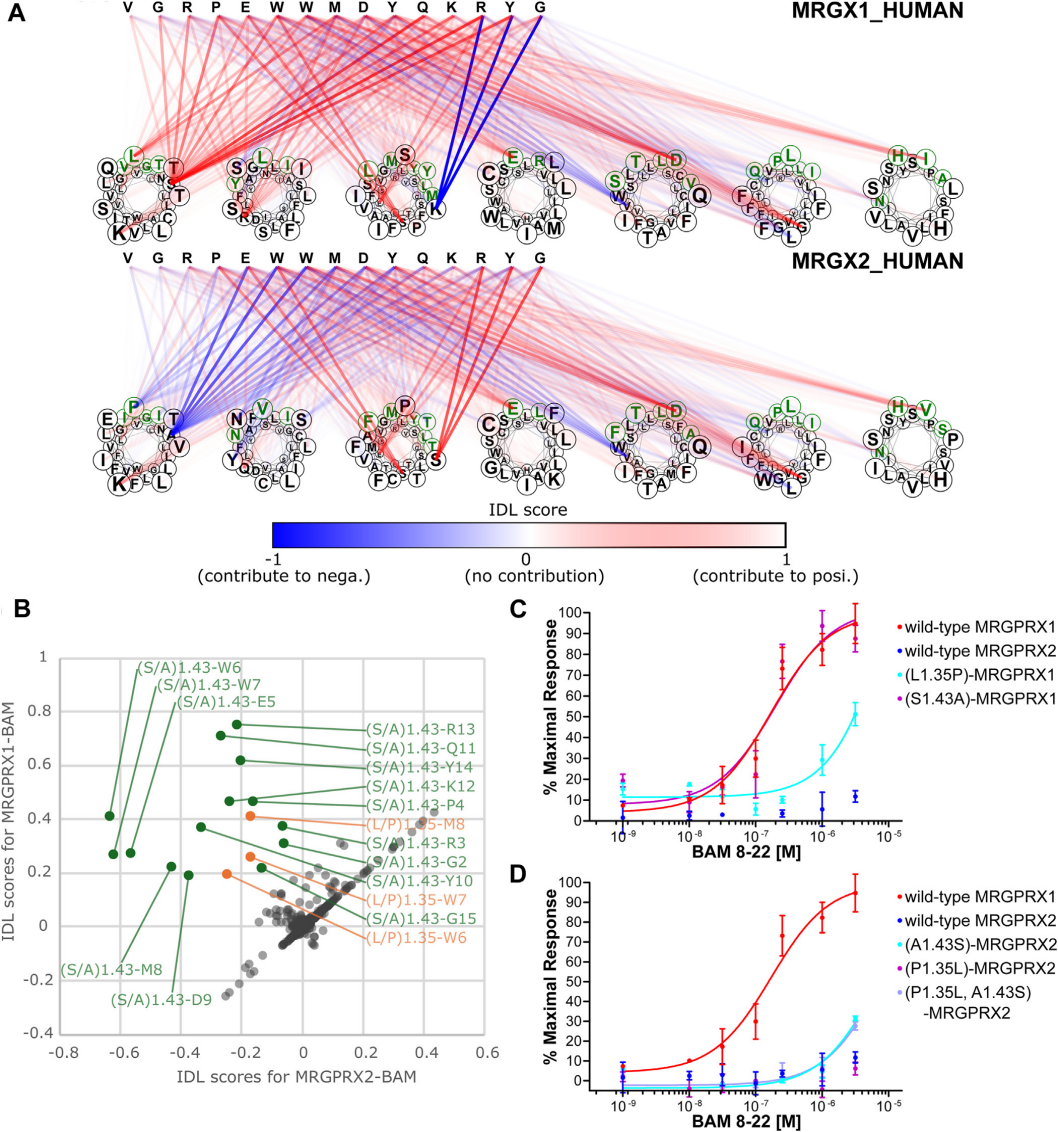
**Estimated IDL scores and DCE validation of the MRGPRX1–BAM pair and MRGPRX2–BAM pair.***A*, IDL scores of the MRGPRX1–BAM pair and MRGPRX2–BAM pair. Transmembrane residues are plotted with *circles*, and cavity-exposed residues are highlighted with *green*. Contribution scores for each peptide and GPCR residue pair are displayed in the heatmap. *B*, scatter plot of IDL scores for the MRGPRX2–BAM pair and MRGPRX1–BAM pair. MRGPRX1-specific high-scoring residues are plotted in *orange* and *green* based on receptor residue position, respectively. *C*, dose-dependent responses of MRGPRX1 mutants to BAM 8–22. *D*, dose-dependent responses of MRGPRX2 mutants to BAM 8–22. BAM, bovine adrenal medulla peptide 8–22; DCE, determinant candidate-exchanged; IDL, interaction determinant likelihood.

We also estimated IDL scores for BAM8–22–MRGPRX1 and BAM8–22–MRGPRX2 as examples of an active CPI and an inactive CPI pair, respectively (Fig. 3*A*). The comparison of IDL scores for BAM8–22–MRGPRX1 and BAM8–22–MRGPRX2 identified L1.35 and S1.43 as MRGPRX1-specific high-scoring residues interacting with Q11 to Y14 of BAM8–22 (Fig. 3*B*, *upper left area*), suggesting that these residues play a major role in the interaction of MRGPRXs with BAM8–22.

Subsequently, we generated MRGPRX1 DCE mutants by replacing L1,35 and A1.43 in MRGPRX1 with the corresponding residues P1.35 and S1.43 in MRGPRX2 and evaluated their pharmacological properties.

The (S1.43A)-MRGPRX1 mutant showed a dose response to BAM8–22 similar to that of wildtype MRGPRX1 (Fig. 3*C* and Table 2). In contrast, the response of the (L1.35P)-MRGPRX1 mutant to 3 μM BAM8–22 decreased to 48.48 ± 1.08% of that of wildtype MRGPRX1 (Fig. 3*C*), indicating the importance of I1.35 in the interaction with BAM8–22. Subsequently, we introduced MRGPRX1-type DCE mutations into MRGPRX2 (P1.35L, A1.43S, and a double mutant; Fig. 3*D* and Table 2). Like wildtype MRGPRX2, the (S1.43A)-MRGPRX2 mutant showed no response to BAM8–22 (Fig. 3*D* and Table 2). Notably, MRGPRX2-P1.35L and the (P1.35L, A1.43S)-MRGPRX2 double DCE mutant showed 30.23 ± 1.08% and 26.53 ± 1.91% responses to 3 μM BAM8–22, respectively (Fig. 3, *C* and *D*). Altogether, we concluded that L1.35 was a critical determinant for the interaction of MRGPRX1 with BAM8–22.

**Table 2:** EC_50_ and the maximal response of MRGPRX1/2 mutants to BAM8–22

GPCR	LogEC_50_ (EC_50_)	Maximal response
MRGPRX1 WT	−6.77 ± 0.16 (0.17 μM)	100.00 ± 7.78%
WT MRGPRX2	ND	ND
(L1.35P)-MRGPRX1	−5.50 ± 0.11 (3.16 μM)	110.00 ± 5.70%
(S1.43A)-MRGPRX1	−6.70 ± 0.20 (0.20 μM)	102.20 ± 9.64%
(A1.43S)-MRGPRX2	−5.15 ± 0.08 (7.08 μM)	103.80 ± 3.17%
(P1.35L)-MRGPRX2	ND	ND
(P1.35L, A1.43S)-MRGPRX2	ND	ND

ND, not determined.

Values are expressed as the means ± standard errors of three replicate analyses, and values that were not determined are expressed as ND.

### Molecular phylogenetic analysis of MRGPRX family GPCRs

**Figure 4:**
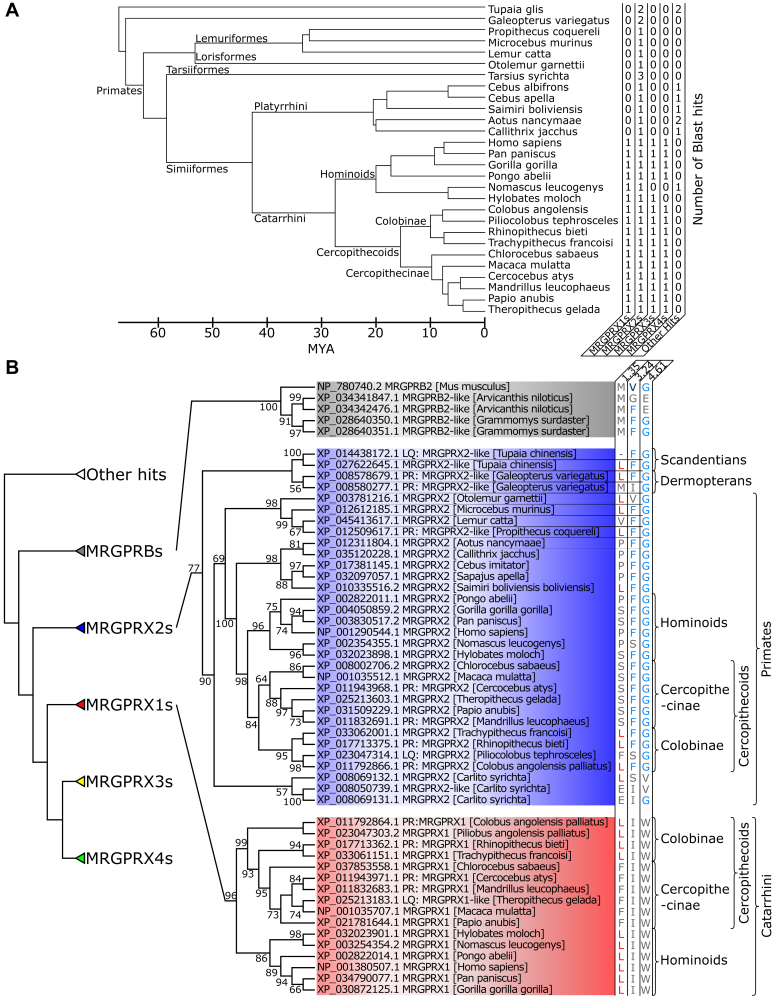
**Time trees of primates and phylogenetic tree of MRGPRXs.***A*, time trees of primates and numbers of orthologs of MRGPRX1–4. The branch length was obtained from the Newick tree of Springer *et al* (16)*.B*, phylogenetic tree of MRGPRXs. The schematic of the gene tree of MRGPRXs was constructed based on the three molecular phylogenetic trees using ORTHOSCOPE. The monophyly-supported and monophyly-unsupported gene clades are indicated by *closed triangles* and *open triangles*, respectively. Clusters for MRGPRX1s and MRGPRX2s were highlighted in *red* and *blue*, respectively. The phylogenetic tree of all taxa is shown in Fig. S6.

Primate-specific MRGPRXs (28) and rodent-specific MRGPRA–Cs (8) are classified into different clades, suggesting that MRGPRXs were duplicated and diversified after the divergence of primates and rodents (28). To explore the evolutionnary lineages for the ligand selectivity of the MRGPRXs and other related GPCRs, we collected 288 homologous genes by using the BLAST on the RefSeq protein database (29) using human MRGPRX1 and two sequences as queries (Fig. 4*A*). Then, the molecular phylogenetic tree was constructed using maximum-likelihood analysis according to the ORTHOSCOPE method (30).

The molecular phylogenetic tree clustered human MRGPRX2 with not only 27 primate genes but also two Scandentia and two Dermaptera genes (Fig. 4*B*, *blue cluster*). Euarchonta, encompassing Primates, Scandentia, and Dermaptera, branched from a common ancestor with Glires, which includes rodents and rabbits, 73.7 to 97.4 million years ago (MYA) (Fig. 4*A*) (31). These results suggested that MRGPRX2 had evolved from a gene belonging to a common ancestor of Euarchonta. Moreover, no orthologs for MRGPRX1, 3, or 4 were found in Scandentia and Dermaptera, indicating that MRGPRX2 was the original MRGPRX gene and that MRGPRX1 evolved from MRGPRX2 in a common ancestor of primates. Furthermore, the distribution of F3.24 and G4.61, which are critical for the interaction of MRGPRX2 with SP (Fig. 2), demonstrated that residues F3.24 and G4.61 were conserved in 27 of 32 MRGPRX2s, including Scandentia and Dermoptera MRGPRX2 (Fig. 4*B*, *blue residues*). Together, these results indicated that MRGPRX2 was generated in Euarchonta 73.7 to 97.4 MYA (Fig. 4*A*) (31) and that MRGPRX2 of the Euarchonta common ancestor acquired the ability to selectively bind to SP. Furthermore, we explored the mouse SP-interacting MRG family gene MRGPRB2 (Fig. 4*B*, *gray cluster*), which is a functional counterpart of MRGPRX2 (18). However, the residues involved in the MRGPRX2-SP and NK1R (F/Y3.24 and G4.61) interactions were not conserved in MRGPRB2, and the corresponding residues in MRGPRB2 were V3.24 and G4.61, indicating that MRGPRB2 acquired the ability to bind to SP in a different manner from MRGPRX2 and NK1R.

MRGPRX1 was clustered with 15 genes (Fig. 4*B*, *red cluster*). Interestingly, MRGPRX1 was present in Catarrhini (Cercopithecidae and Hominidae) (Fig. 4*B*). The Catarrhini were considered to have branched from a common ancestor with Platyrrhini 33.55 to 49.48 MYA and then diverged into Catarrhini, at 19.67 to 32.83 MYA (Fig. 4*A*) (16). These phylogenetic relationships of Catarrhini demonstrate that the MRGPRX1 family genes diverged at 33.55 to 49.48 MYA from a common ancestor of Catarrhini (16). Subsequently, we analyzed the distribution of L1.35, which is the critical residue for BAM8–22 binding to human MRGPRX1 (Fig. 4*B*, *red residues*). Of particular interest is that *Otolemur*, *Microcebus*, *Propithecus*, *Saimiri*, *Trachypithecus*, *Rhinopithecus*, and *Colobus* MRGPRX2s possess both L1.35 (BAM8–22 determinant) and F3.24 and G4.61 (SP determinants) (Fig. 4*A*, *blue residues*), suggesting that these MRGPRX2s interact with both BAM8–22 and SP. Furthermore, we analyzed the conservation pattern of L1.35, the critical residue for BAM8–22 activity, in MRGPRX1 cluster genes. The Lue residues of Hominidae MRGPRX1s corresponding to L1.35 in human MRGPRX1 were conserved, but Cercopithecidae MRGPRX1s consisted of L1.35-type conserved Colobinae MRGPRX1s and F1.35-type Cercopithecinae MRGPRX1s (Fig. 4*C*, *red cluster*). These results suggest that MRGPRX1 acquired the ability to selectively bind to BAM8–22 at least 19.67 to 32.83 MYA in a common ancestor of Hominidae.

**Figure 5:**
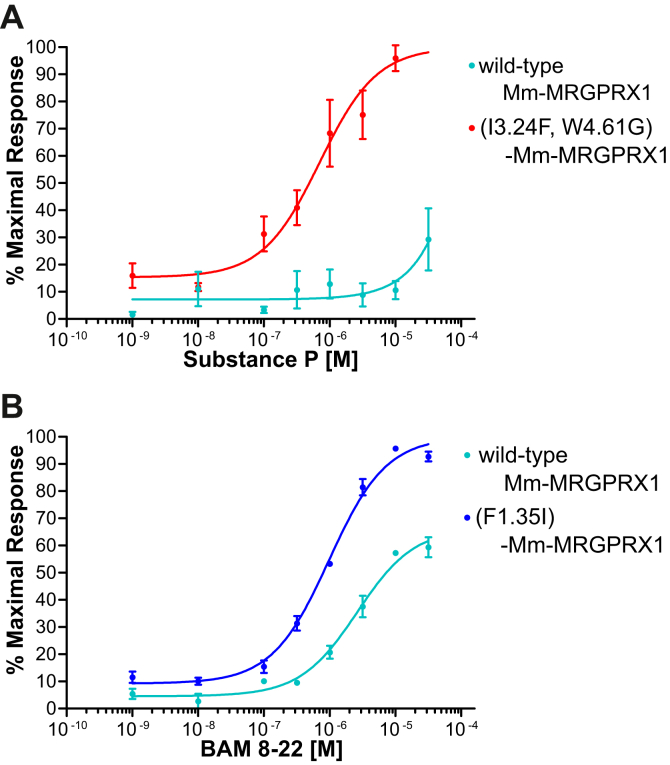
**Dose-dependent responses of Mm-MRGPRX1 mutants to BAM 8–22 and SP.***A*, the dose-dependent responses of wildtype Mm-MRGPRX1 and (F1.35I)-Mm-MRGPRX1 mutant, which has a determinant residue for BAM 8–22, to BAM8-22 are shown in cyan and blue, respectively. *B*, the dose-dependent responses of wildtype Mm-MRGPRX1 and (I3.24F, W4.61G)-Mm-MRGPRX1 mutant, which has determinant residues for SP, to SP is shown in *cyan* and *red*, respectively. BAM, bovine adrenal medulla peptide; Mm-MRGPRX1, Macaca mulatta MRGPRX1; SP, substance P.

The comparison of determinant residues among MRGPRX homologs suggested that MRGPRX1s of Colobinae including *Macaca mulatta* fail to significantly interact with neither BAM8–22 nor SP because of mutation of L1.35F and mutation of F3.24I and G4.61W. To further validate the aforementioned evolutionary scenario, we evaluated the pharmacological properties of *M. mulatta* MRGPRX1 (Mm-MRGPRX1) and the determinant-recovering mutants of Mm-MRGPRX1, namely (F1.35L)-MRGPRX1 for BAM-8–22 and (F3.24I, G4.61W)-MRGPRX1 for SP (Fig. 5 and Table 3). The intact Mm-MRGPRX1 showed a dose response to BAM8–22 with an EC_50_ of 2.57 μM, which was 15-fold less potent affinity than that of human MRGPRX1. Notably, (F1.35L)-Mm-MRGPRX1 mutant showed a dose response to BAM8–22 in EC_50_ of 0.95 μM (Fig. 5*A* and Table 3). Moreover, the Emax value was also enhanced 1.5-fold in (F1.35L)-Mm-MRGPRX1, compared with intact Mm-MRGPRX1. These signaling assays using the determinant-recovering mutants proved that L1.35 in native Mm-MRGPRX1 reduced the interaction with BAM8–22 in Cercopithecinae. Furthermore, we also evaluated the SP recognition of Mm-MRGPRX1. Although the intact Mm-MRGPRX1 exhibited no response to SP, (F3.24I, G4.61W)-Mm-MRGPRX1 showed a dose response to SP with an EC_50_ of 0.20 μM (Fig. 5*B* and Table 3). Consequently, these results provide evidence that the generation of the F3.24 and G4.61 was crucial for the acquisition of the SP recognition by MRGPRX1 during the evolution of Catarrhini. Collectively, the present IDL score–based studies verified not only the interaction determinant residues but also the evolutionary lineages of the ligand selectivity of MRGPRX1 and MRGPRX2 at the molecular level.

**Table 3:** EC_50_ and the maximal response of Mm-MRGPRX1 mutants to BAM8–22 and SP

Ligand	GPCR	LogEC_50_ (EC_50_)	Maximal response
BAM8–22	Mm-MRGPRX1 WT	−5.59 ± 0.08 (2.57 μM)	66.54 ± 2.91%
	(F1.35L)-Mm-MRGPRX1	−6.02 ± 0.05 (0.95 μM)	100.00 ± 1.94%
SP	Mm-MRGPRX1 WT	ND	ND
	(I3.24F, W4.61G)-Mm-MRGPRX1	−6.16 ± 0.14 (0.69 μM)	100 ± 5.28%

ND, not determined.

Values are expressed as the means ± standard errors of three replicate analyses, and values that were not determined are expressed as ND.

## Discussion

Neuropeptides play multiple biological roles, mainly by activating their cognate GPCRs, and the ligand specificity of GPCRs is regulated by the multiple amino acid residues that are responsible for ligand interactions. To date, elucidating key amino acid determinants for specific interactions involves point mutagenesis. However, this research strategy necessitates the generation of numerous amino acid–replaced mutants, making it impractical for comprehensive analysis (*e.g.*, in case where mutants for multiple amino acids are required). These shortcomings restricted the elucidation of the molecular mechanisms underlying peptide–GPCR interactions and their specificity (32), including interactions between MRGPRX and SP or BAM8–22. In this study, we developed the IDL score method for predicting essential amino acid residues for specific interactions of GPCRs and verified the determinant residues by signaling assays for DCE mutants (Fig. 1). Notably, the IDL score method was not specific to the elucidation of determinants for ligand selectivity in MRGPRX1/2. Performance evaluations confirmed their high accuracy in predicting residues involved in peptide recognition for multiple GPCRs, including NTR1, MC4R, CCKBR, GNRHR, C5AR, and V2R, which had the greatest number of experimentally validated mutation records in the GPCRDB (Figs. S3 and S4). These results suggest that the IDL score method is a powerful tool for the identification of various peptide–GPCR interactions. The IDL score estimation predicted the crucial roles of P1.35, F3.24, and G4.61 in the MRGPRX2–SP interaction and those of L1.35 and A1.43 in the MRGPRX1–BAM8–22 interaction (Figs. 2, *A* and *B*, and 3, *A* and *B*), and the subsequent signaling assays of DCE mutants provided experimental evidence that F3.24 and G4.61 are determinants for the interaction of MRGPRX2 with SP (Fig. 2, *C* and *D*) and that L1.35 is a determinant for the interaction of MRGPRX1 with BAM8–22 (Fig. 3, *C* and *D*). Notably, a combination of mutations (P1.35, F3.24, and G4.61) revealed that F3.24 and G4.61 double DCE mutation was responsible for the acquisition of the interaction with SP (Fig. 3). It is also noteworthy that the signaling assays of DCE mutants, rather than the standard approach of random alanine-replaced mutants, led to the verification of the process by which the ligand recognition capacity of MRGPRX1 and MRGPRX2 evolved from that of a common ancestor. Consequently, the present study highlights the marked usefulness of IDL score estimation and DCE mutation analysis in the identification of amino acids responsible for the ligand specificity of orthologous GPCRs.

In general, class A GPCRs interact with ligands at a ligand-binding cavity that is formed by transmembrane helices (33), and the 30 residues exposed on the cavity surface have been defined by alignment (Figs. 2*A* and 3*A*, *green residues*) (34). Moreover, several statistical analyses have been performed with the assumption that these residues were conserved among GPCRs that share the same ligand, suggesting that cavity-exposed residues can serve as determinants of ligand–receptor physical interactions and of the ligand selectivity of GPCRs (35). Consistently, L1.35, a determinant of the interaction of MRGPRX1 with BAM8–22, is a cavity-exposed residue (Fig. 3*A*, *green residues*), which supports the idea that this residue is involved in ligand–receptor physical interactions. On the other hand, the key residues of MRGPRX2 that interact with SP, F3.24, and G4.61, are not located in the ligand-binding cavity (Fig. 2*A*, *black residues*), indicating that these residues play crucial roles in the determination of ligand affinity in an indirect manner rather than directly binding to SP. Concerning nonpeptidergic GPCRs, point mutation analyses of the dopamine receptor DRD2 showed that both cavity-exposed residues and nonexposed residues of DRD2 are involved in serotonin or dopamine responses (32). Furthermore, mutations of cavity-exposed residues were shown to alter the potency of the response of DRD2 to serotonin and dopamine, whereas mutations of nonexposed residues were shown to increase or decrease only the maximal efficacy of the ligands but not the potency. In contrast, the present study verified that the potency of the effect of BAM8–22 on the cavity-exposed residue mutant (P1.35L)-MRGPRX2 was not increased compared with the potency with wildtype MRGPRX1 (Fig. 3, *A* and *C* and Table 1) and that SP exhibited comparable potency with the nonexposed residue mutant (I3.24F and W4.61G)-MRGPRX1 and wildtype MRGPRX2 (Fig. 2*D*). These results suggest that the key residue positions (cavity-exposed or nonexposed residues) related to ligand affinity vary among GPCRs.

The present study also highlighted different features in the contribution of residues to ligand specificity. The (I3.24F, W4.61G)-MRGPRX1 double DCE mutant and the (L1.35P, I3.24F, W4.61G)-MRGPRX1 triple mutant displayed SP activity with EC_50_ values comparable to that of MRGPRX2-SP, whereas (L1.35P, W4.61G)-MRGPRX1 and (L1.35P, I3.24F)-MRGPRX1 double mutants failed to exhibit any response to SP (Fig. 2*D* and Table 1). Furthermore, both (F3.24I)-MRGPRX2 and (G4.61W)-MRGPRX2 single mutants also failed to exhibit any response to SP, although the membrane expression was comparable to that of wildtype MRGPRX2 (Figs. 2 and S5). These results provide evidence that the presence of both F3.24 and G4.61 is essential for the specific interaction of MRGPRX2 with SP. In other words, the binding of MRGPRX1 and -2 to SP is determined by a synergistic effect of the essential residues F3.24 and G4.61. Furthermore, the SP determinants were conserved in NK1R (Y3.24 and G4.61), reinforcing the importance of these residues as determinants of the interaction of GPCRs with SP. In addition, we confirmed that Mm-MRGPRX1, which exhibited no response to SP in its native form, also responds to SP by mutating F3.24 and G4.61 (Fig. 5). These results suggest that SP recognition by the synergistic effects of F3.24 and G4.61 is a common mechanism among primates. In contrast, both the MRGPRX2-type mutant (P1.35L)-MRGPRX1 and the MRGPRX1-type mutant (L1.35P)-MRGPRX2 responded to BAM8–22 with lower potency than wildtype MRGPRX1 (Fig. 3*D* and Table 2). These mutation analyses suggested that the response to BAM8–22 was not determined in a synergistic manner but rather additively. We also showed that Mm-MRGPRX1, which has a P1.35L-type mutation in the intact sequence, responds to BAM8–22 with similar potency to (P1.35L)-MRGPRX1, whereas (L1.35P)-Mm-MRGPRX1 responds to BAM8–22 with a higher Emax and potency. However, the EC_50_ of (L1.35P)-Mm-MRGPRX1 against BAM8–22 was 0.95 μM, which is less potent than that of human MRGPRX1 (0.17 μM). This suggests that P1.35L and other loss-of-activity mutations in Mm-MRGPRX1 additively weaken its potency to BAM8–22. Collectively, the GPCR residues participating in ligand specificity can be classified into at least two types: synergistic-type residues (F3.24 and G4.61) are nonexposed, whereas the additive-type residue (P1.35) is a cavity-exposed residue (Figs. 2 and 3). Overall, the present study suggests the unprecedented hypothesis that a nonexposed residue accommodatively contributes to ligand recognition *via* the cavity backbone geometry, and a surface-exposed residue additively participates in ligand recognition *via* modulation of the cavity side-chain shape.

**Figure 6:**
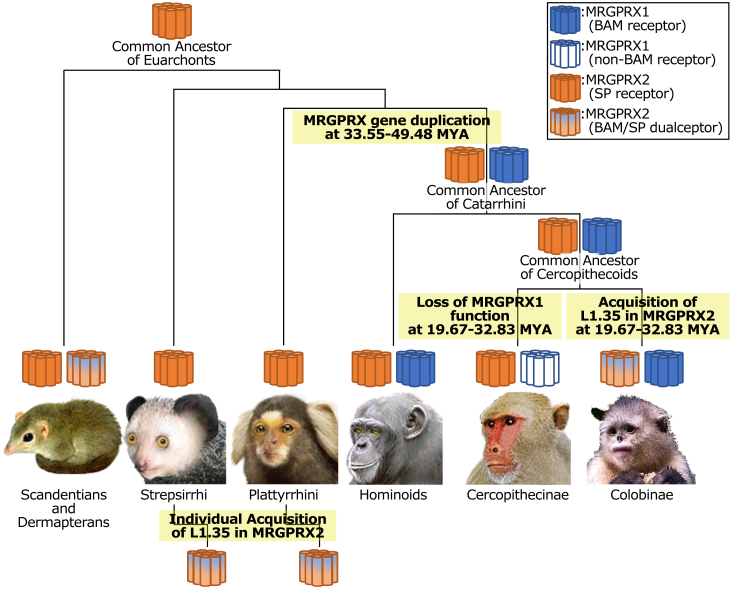
**The timing of the duplication of MRGPRX1 and MRGPRX2 and the estimated age at which each acquired the ability to recognize BAM8–22 and SP.** The images of animals are sourced from TogoTV (https://togotv.dbcls.jp/en, CC-BY-4.0) and Rod Waddington from Kergunyah, Australia (CC BY-SA 2.0 *via* Wikimedia Commons). BAM, bovine adrenal medulla peptide; SP, substance P.

Canonical molecular phylogenetic tree analyses and related evolutionary statistical analyses cannot be used to investigate the evolutionary processes of the acquisition and diversification of selective ligand recognition by receptors, because these methods are based on the similarity of sequences and functions and on the hypothesis that the amino acid residues involved in a common function in orthologous proteins, including selective ligand recognition, are conserved. In contrast, the present IDL score method identified L1.35 as the key residue for the selective recognition of BAM8–22 by MRGPRX1, leading to the presumption that MRGPRX1 of the Catarrhini common ancestor recognized BAM8–22, but Cercopithecinae MRGPRX1s lost interaction with BAM8–22 19.67 to 32.83 MYA because of the mutation at L1.35 (Fig. 6). This estimation is also consistent with the decreased potency of Mm-MRGPRX1 for BAM8–22, whereas the L1.35P mutation to Mm-MRGPRX1 markedly potentiated the activity of BAM8–22 (Fig. 5). Likewise, the key residues for SP recognition (F3.24 and G4.61) were conserved in Euarchonta MRGPRX2s, leading to the presumption that MRGPRX2 recognized SP in the Euarchonta common ancestor 73.7 to 97.4 MYA (Fig. 6). Moreover, the SP determinant residue is conserved only in GPCRs of the MRGPRX2 clade and not in GPCRs of the MRGPRX1 clade, indicating that SP recognition has been conserved as a crucial function within the MRGPRX2 family. Notably, *Otolemur*, *Microcebus*, *Propithecus*, *Saimiri*, *Trachypithecus*, *Rhinopithecus*, and *Colobus* MRGPRX2s were suggested to recognize both BAM8–22 and SP because of the acquisition of L1.35 (Fig. 4, *red residue*). The distribution of such dual ligand recognition-type MRGPRX2 in multiple phylogenetic clusters strongly suggests that L1.35 might have been acquired by MRGPRX2 in individual species beginning 30 MYA. To our knowledge, this is an unprecedentedly high frequency of ligand specificity–related mutations. Also, of interest is whether other receptors have also altered or acquired ligand specificity by such mutations in their evolutionary lineages. Furthermore, the present study showed that some species-specific functional selection pressure arises from the binding of MRGPRX2 to BAM8–22. The interaction of MRGPRX2 with SP induces inflammatory itch and pain (11, 12, 13), and the interaction of MRGPRX1 with BAM8–22 is involved in the induction of itch and the suppression of pain signaling in humans (9, 10). Collectively, these findings suggest that the differences in the ligand-specificity pattern of MRGPRX2 are responsible for the species-specific biological importance of inflammatory and nociceptive signaling among primates.

The IDL score analysis also demonstrated the mode of recognition of other SP receptors. The key residues for SP recognition were conserved in NK1R as Y3.24 and G4.61 (Fig. 2*E*). The I3.24F, W4.61G double-mutated Mm-MRGPRX1 was also activated by SP because of the, suggesting that SP recognition by F3.24, G4.61 is widespread in GPCRs. However, the corresponding residue in mouse MRGPRB2 was mutated to V3.24 (Fig. 4). This conservation and variation of determinant amino acids suggests that MGRX2 and NK1R share the common recognition mode of SP regardless of their molecular phylogenetic unrelatedness, whereas MRGPRB2 recognizes SP by a different molecular mechanism from that of MRGPRX2 despite their orthology. Collectively, these results also highlighted the prominent advantages of IDL score analysis as the only method that can estimate the divergence age for the acquisition and loss of ligand recognition for both orthologous and phylogenetically distant GPCRs. Recently, 3D descriptors for GPCRs were constructed from molecular dynamics data to identify key residues in ligand recognition, which cannot be determined from the crystal structure or the predicted structure of AlphaFold (36). However, this method was restricted to GPCRs with high-resolution crystal structures, which hinders the analysis of most orthologs and paralogs. A structure-free method, AlphaMissense (37), which predicts mutation-disease correlation using mutation information in genome-wide association study data, is used only for human disease–related mutations. When predicting ligand determinants of GPCRs, even human GPCRs, which interact with multiple endogenous ligands, such as MC4R, many of the mutations associated with the predicted etiology are likely to be those that do not contribute to the target ligand–receptor pair. Indeed, the prediction accuracy of inactivating mutations by IDL score was higher than that of inactivating mutations by AlphaMissense (see Supporting Information). These findings also reinforce the IDL score method as the only procedure that can identify various evolutionary lineages of ligand–GPCR interactions.

In conclusion, we systematically elucidated multiple determinants for peptide recognition by GCPRs and their evolutionary lineages using a combination of original machine learning–based IDL scores, DCE validation, and phylogenetic analyses of two homologous GPCRs, MRGPRX1 and -2. This is the first systematic identification of determinants for ligand–GPCR recognition and its evolution.

## Experimental procedures

### PD-incorporated SVM and IDL score calculation

PD-incorporated SVM was constructed according to our previous study (23, 38). Here, we used same 2467 CPIs, which included 932 human interactions, 384 mouse interactions, 568 other vertebrate interactions, and 581 invertebrate interactions, from the IUPHAR Database (39), the UniProtKB Knowledgebase (40) and the literature as in our previous study (38). Then, an equivalent number of noninteracting pairs (nonZCPIs) was generated by swapping the GPCRs and peptides of CPIs. The peptides and GPCRs in the collected CPIs were converted to PDs (23) and TM z-scale descriptors (41), and each CPI and non-CPI was represented as a linearized-outer product of the peptide and GPCR descriptors. This operation is equivalent to creating a descriptor that combines elements of each GPCR and PD. With these descriptor-converted CPIs and non-CPIs, a linear SVM including genetic algorithm–based feature selection (23, 38) was trained to generate a prediction model. The prediction model outputs the scaled distances from the hyperplane of the trained model and takes values from minus infinity (non-CPI) to plus infinity (CPI). Since the prediction model was constructed with linear SVMs, the distances from the hyperplane (*dist*) were represented as follows:

(1) $$dist=\sum_{i}\sum_{j}w_{i,j}*{pD}_{i}*{gD}_{j}$$

where *pD*_*i*_ represents the ith element of the PD, and *gD*_*i*_ represents the jth element of the GPCR descriptor. The trained weight for the combination descriptor of *pD*_*i*_ and *gD*_*i*_ was denoted *wi*_*,j*_. Since each PD element represents the existence of 5-length amino acid motifs, the IDL score for the Nth peptide residue can be defined by collecting the motifs containing Nth peptide residues as follows:

(2) $$IDLscore\;\left(N\right)=\sum_{\begin{array}{c} i=motifs\;containing\\ Nth\;peptideresidue\; \end{array}}\sum_{j}w_{i,j}*{pD}_{i}*{gD}_{j}$$

In TM-z scale descriptors, each residue in the transmembrane region was converted to 5-length numerical vectors (41), and the 5M-4 to 5Mth elements of the descriptors explicitly included the Nth transmembrane residue. Thus, the IDL score of the Mth GPCR residue can be defined as follows:

(3) $$IDLscore\;\left(M\right)=\sum_{i}\sum_{j=5M-4}^{5M}w_{i,j}*{pD}_{i}*{gD}_{j}$$

By combining these strategies, the IDL score of the pair of peptide and GPCR residues can be defined as follows:

(4) $$IDLscore\;\left(N,M\right)=\sum_{\begin{array}{c} i=motifs\;containing\\ Nth\;peptideresidue\; \end{array}}\sum_{j=5M-4}^{5M}w_{i,j}*{pD}_{i}*{gD}_{j}$$

### Plasmid construction

ORFs of MRGPRX1 and MRGPRX2 purchased from OriGene and Mm-MRGPRX1 synthesized in Genscript were C-terminally fused with the human Gαq_16_ protein as described previously (23). Briefly, the human Gαq_16_ ORF clone (OriGene) was amplified and ligated into the XbaI site of a pcDNA6 plasmid (Invitrogen). Then, MRGPRX1 and MRGPRX2 were subcloned (Table S1) into the NotI–XbaI site of the Gαq_16_-ligated pcDNA6 plasmids. MRGPRX1 and two mutants were constructed using the PrimeSTAR Mutagenesis Basal Kit with mutation oligos (Table S1).

### Cell culture and calcium accumulation assay

COS-7 cells were purchased from American Type Culture Collection and cultured in Dulbecco's modified Eagle's medium (Nacalai Tesque) supplemented with 10% fetalbovine serum (Sigma) and maintained at 37°C under 5% CO_2_. Intracellular Ca^2+^ mobilization was measured as previously described (42, 43). In brief, 5 × 10^5^ COS-7 cells were spread on a 60-mm dish 1 day before transfection. About 10 μg of GPCR expression vector were transfected into the cells using Lipofectamine 2000 (Thermo Fisher Scientific) according to the manufacturer’s instructions. After the 24-h incubation, 6 × 10^4^ cells per well were respread on a 96-well plate for the following assay. Each MRGPRX mutant fused with human Gαq_16_ for expression at the cell membrane was confirmed by immunostaining using the anti-Gαq_16_ antibody (Ori Gene TA318890) (Fig. S5). SP (PEPTIDE INSTITUTE) and BAM8–22 (Tocris Bioscience) were used for the assays. Real-time Ca^2+^ mobilization of Gαq_16_-fused MRGPRX1/2 was measured using a Ca5 kit (Molecular Devices) and FlexStation III according to the manufacturer’s instructions. The calcium accumulation data were analyzed using Prism, version 6 (GraphPad) and found to fit a sigmoidal concentration–response curve, and the means ± SEMs of EC_50_ were calculated.

### Molecular phylogenetic analysis of MRGPRX-family GPCRs

The sequences of human MRGPRX1–4 were used as a BLASTP search query against the RefSeq protein database, and the hit sequences were screened using an E-value cutoff of <10^−3^. The transmembrane sequences were searched using GPCRalign (44), and sequences without residues 1.35 (N terminal of the conserved transmembrane region) to 7.51 (C terminal of the conserved transmembrane region) in Ballesteros–Weinstein generic numbers were eliminated as partial sequences. From the remaining full-length sequences, the top five hits were aligned using CLUSTAL Wmpi 0.13 (45), and maximum-likelihood phylogenetic trees based on a JTT matrix–based model (46) with 100 bootstraps were created using Fast Tree 2.1.10 (47). The constructed phylogenetic trees were drawn using MEGA X software (48).

## Data availability

All data in this study are presented in the main article and the supporting information.

## Supporting information

This article contains supporting information (23, 25, 26, 38, 49, 50, 51, 52).

## Conflict of interest

The authors declare that they have no conflicts of interest with the contents of this article.
