# Supplementary material for: Evolutionary scenarios for the specific recognition of nonhomologous endogenous peptides by G protein–coupled receptor paralogs
Source: J Biol Chem. 2024 Dec 25;301(2):108125. doi: 10.1016/j.jbc.2024.108125 (PMC11910321; doi:10.1016/j.jbc.2024.108125)
Supplement: Supplemental data [file mmc1.docx]

**Supporting Information**

**Evolutionary Scenarios for the Specific Recognition of Non-homologous Endogenous Peptides by G Protein-Coupled Receptor Paralogs**

Akira Shiraishi^1^, Azumi Wada^1^, and Honoo Satake^1^

^1^ Bioorganic Research Institute, Suntory Foundation for Life Sciences, Kyoto, Japan

*Correspondence: Akira Shiraishi, Bioorganic Research Institute, Suntory Foundation for Life Sciences, 619-0284, Kyoto, Japan. E-mail: [shiraishi@sunbor.or.jp](mailto:shiraishi@sunbor.or.jp)

This document contains six figures (Figs. S1-6) and one table (Table S1)

**Interaction factor estimation and comparison with cocrystallized structure**

Our PD-incorporated SVM was trained with the same dataset of 2,467 compound–protein interactions (CPIs), which are converted to TM-z scale descriptors and peptide descriptors, respectively as in our previous study(1). Then, the interaction prediction scores (ISs) were estimated as the sum of terms (W_x,y_R_x_L_y_ in Fig. S2) , representing the product of TM-z scale descriptor elements (R_x_ in Fig. S2), peptide descriptor elements (L_y_ in Fig. S2) and weight vector elements (W_x,y_ in Fig. S2)(1, 2). These terms explicitly incorporate peptide motifs and GPCR residues as descriptor elements. To estimate the contributions of specific pairs of peptide and GPCR residues to the IS calculation, we defined the IDL score as the sum of the terms including descriptor elements corresponding to the residues of interest (Fig. S2). In IDL score estimation, GPCR residue positions were confined to those with Ballesteros-Weinstein generic numbers(3) due to the TM-z scale method. Since a positive prediction score is defined as corresponding to an interacting pair, the residue pairs with positive IDL scores are considered as the residues responsible for the interaction.

To evaluate the correlation of IDL scores with peptide–GPCR interaction, we compared the IDL scores for the interaction between neurotensin and its cognate receptor, NTR1, using the cocrystallized structures(4). The top five GPCR residues with the highest IDL scores were R311^6.38^, G144^3.27^, R327^6.54^, W130^2.67^, and Y146^3.29^ (Fig. S3A) for interaction with P10-L13 of neurotensin residues. Of those, the cocrystallized structures demonstrated that Y146^3.29^ (IDL score=0.633) and R327^6.54^ (IDL score=0.468) formed hydrogen bonds with neurotensin (Fig. S3B). Interestingly, the top amino acids included not only the presence of L13 at the C-terminus but also the motif PYIL at P10-L13 (Fig. S3A). This PYIL was motif was shown to be conserved among neurotensin and neuromedin Ns in vertebrates(5) and suggested to interact with van der Waals interactions between PYIL and NTSR1 by cryo-electron microscopy structures of the complex(6), highlighting the importance of residues with high IDL scores to the interaction. On the other hands, R311^6.38^-I12 had the highest (0.998) IDL score and a 29-Å distance (Fig. S3C). G144^3.27^-P10 (IDL score=0.450) and W130^2.67^-P10 (IDL score=0.514) had distances of 18 Å and 14 Å, respectively, indicating no physical interaction of the R311^6.38^-I12, G144^3.27^-P10 and W130^2.67^-P10 pairs (Fig. S3C). However, R311^6.38^ was suggested to form a water-mediated hydrogen bond with the backbone of N257^5.58^ in the MD simulation(7), supporting the indirect effects implied by the IDL score. Furthermore, G144^3.27^ and W130^2.67^, both of which are helix-breaking residues, exhibited high IDL scores. These high-IDL score residues suggest that these helical breakers destabilize the helix and control the size of the binding pocket to allow neurotensin to bind. Collectively, the original IDL score-based prediction was shown to identify amino acid residues of neurotensin and NTS1 that are crucial for crystallographically validated physical interactions and distal (indirect) effects on interaction.

For further evaluation, we compared the GPCR residues with high IDL scores to the experimentally identified peptide recognition of mutant GPCRs. First, we collected information on the interaction changes of five peptidergic GPCRs (C5AR1, V2R, MC4R, GNRHR, CCKBR) with the greatest number of literatures on mutations included in the GPCRDB. We defined interaction-decreasing mutations as those with Emax and Bmax values less than half of intact GPCRs or with the EC50, IC50, and Kd values more than tenfold higher than those of intact GPCRs. Conversely, activity-unrelated mutations were defined as those with Emax and Bmax values exceeding 80% of intact GPCRs and the EC50, IC50, and Kd values less than twice those of intact GPCRs. Under these criteria, we collected a total of 130 interaction-decreasing mutations (50 for GnRHR, 35 for MC4R, 23 for C5AR1, 11 for CCKBR, and 11 for V2R) and 99 activity-unrelated mutations (27 for GnRHR, 46 for MC4R, 8 for C5AR1, 10 for CCKBR, and 8 for V2R).

We then calculated the difference in IDL scores for each GPCR-peptide residue pair between intact and mutant GPCRs. Since mutation experiments could not determine which peptide residues interacted with the mutated GPCR residues, the smallest IDL score change across all peptide residues was considered as the IDL score for each GPCR residue. The performance of the IDL score method for predicting interaction-decreasing mutations was evaluated using the area under the receiver operating characteristic curve (AUC), resulting in an AUC of 0.88 (Fig. S4). We also predicted the pathogenicity of these mutations using Alphamissense, which produced an AUC of 0.71 (Fig. S4), which was lower than that of our proposed IDL scores. These results suggest that the IDL-score method can predict amino acids involved in peptide-GPCR interactions across diverse peptides and GPCRs.

We further compared the AUCs for each GPCR to understand the differences in prediction accuracy between the IDL score and Alphamissense in detail (Fig. S4). For MC4R and CCKBR, the IDL score showed higher AUC values (0.94 for MC4R and 0.74 for CCKBR) than Alphamissense (0.67 for MC4R and 0.48 for CCKBR). Both MC4R and CCKBR recognize structurally varied ligands (MSHs, ACTH, agouti-related protein for MC4R, and CCKs and gastrins for CCKBR), and some mutations in these GPCRs affect different ligands differently(8). Therefore, in principle, GPCRs that recognize multiple ligands can only be accurately predicted using the IDL-score method. In contrast, both the IDL score and Alphamissense showed high AUC values for GNRHR (0.84 for the IDL score and 0.83 for Alphamissense), C5AR1 (0.81 for the IDL score and 0.79 for Alphamissense) and V2R (0.89 for the IDL score and 0.86 for Alphamissense) which endogenously recognize only structurally similar ligands, respectively. In this case, determinants and pathogenicity follow a simple rule for a single ligand, making prediction easier for both methods. Collectively, the IDL score-based prediction was shown to identify amino acid residues involved in interactions even if the GPCR recognizes multiple structurally different peptides.


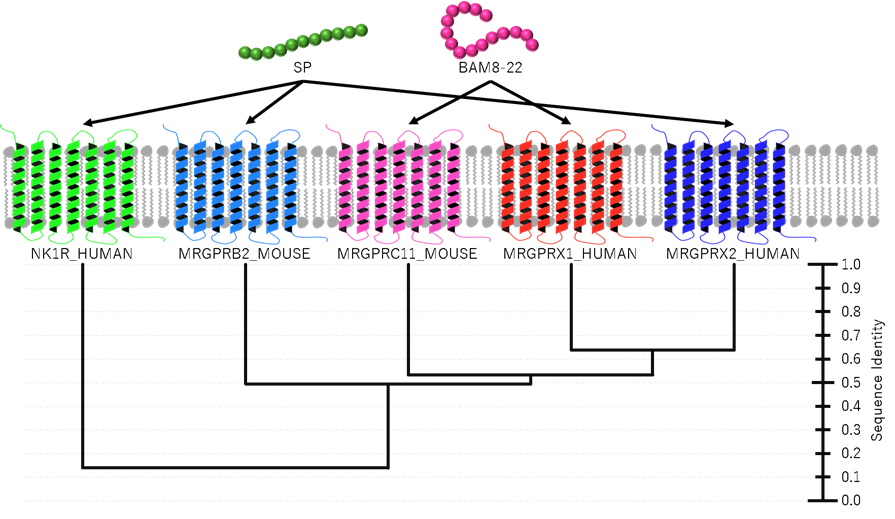


**Figure S1. Receptors for Substance P (SP), those for BAM8-22 and their paralogous genes.** Each arrow indicates the peptide-GPCR interactions. Identity of GPCR sequences is shown with single-linkage clustering.


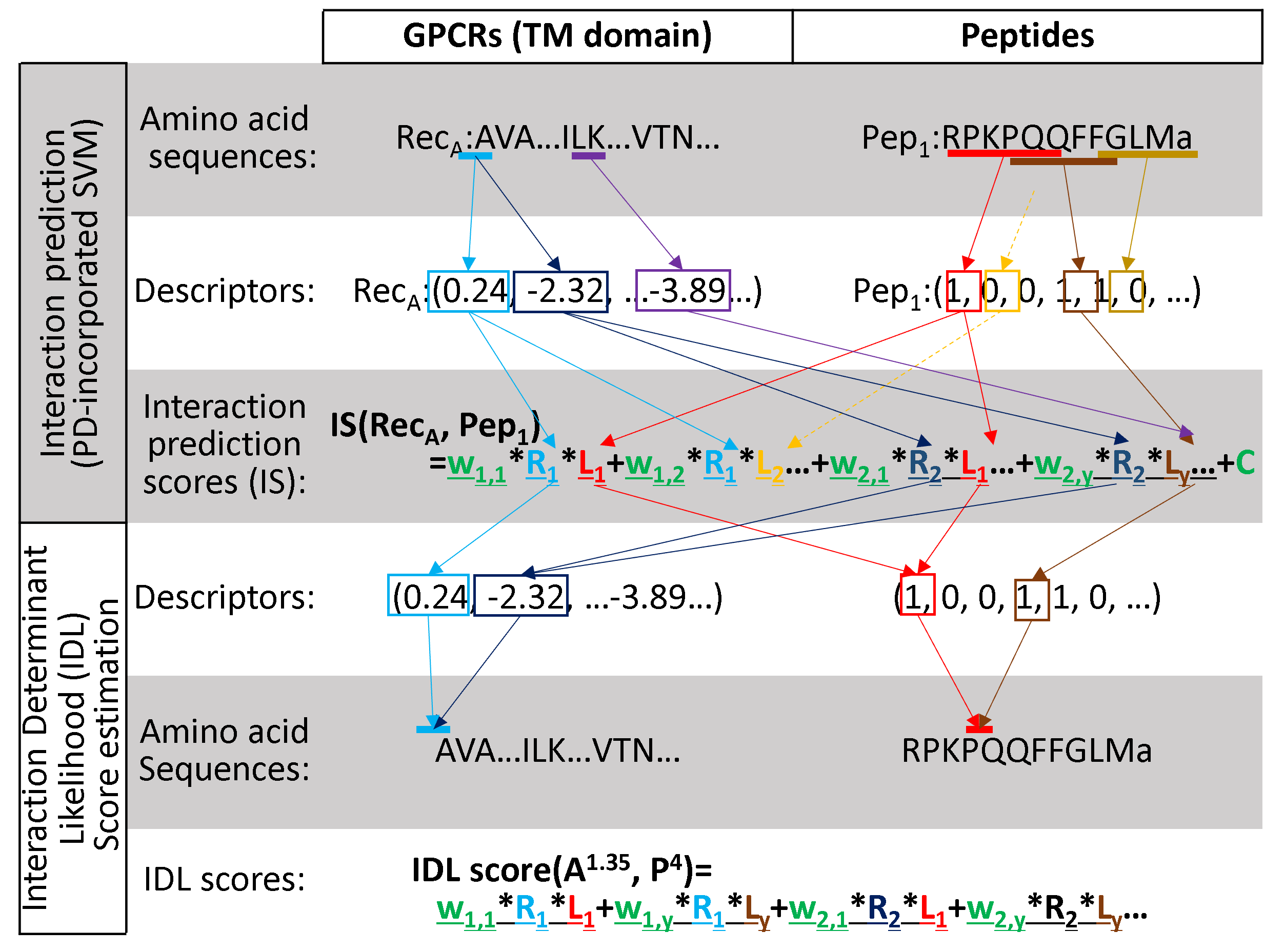


**Figure S2. Prediction flow of PDSVM and estimation flow of contribution scores.** The trained PD-incorporated SVM model estimates interaction prediction scores (ISs), which are the sumproducts of GPCR descriptors, peptide descriptors, and weight vector elements. Here, we defined the interaction determinant likelihood (IDL) score as the sum of the terms including descriptor elements corresponding to the residues of interest.

**
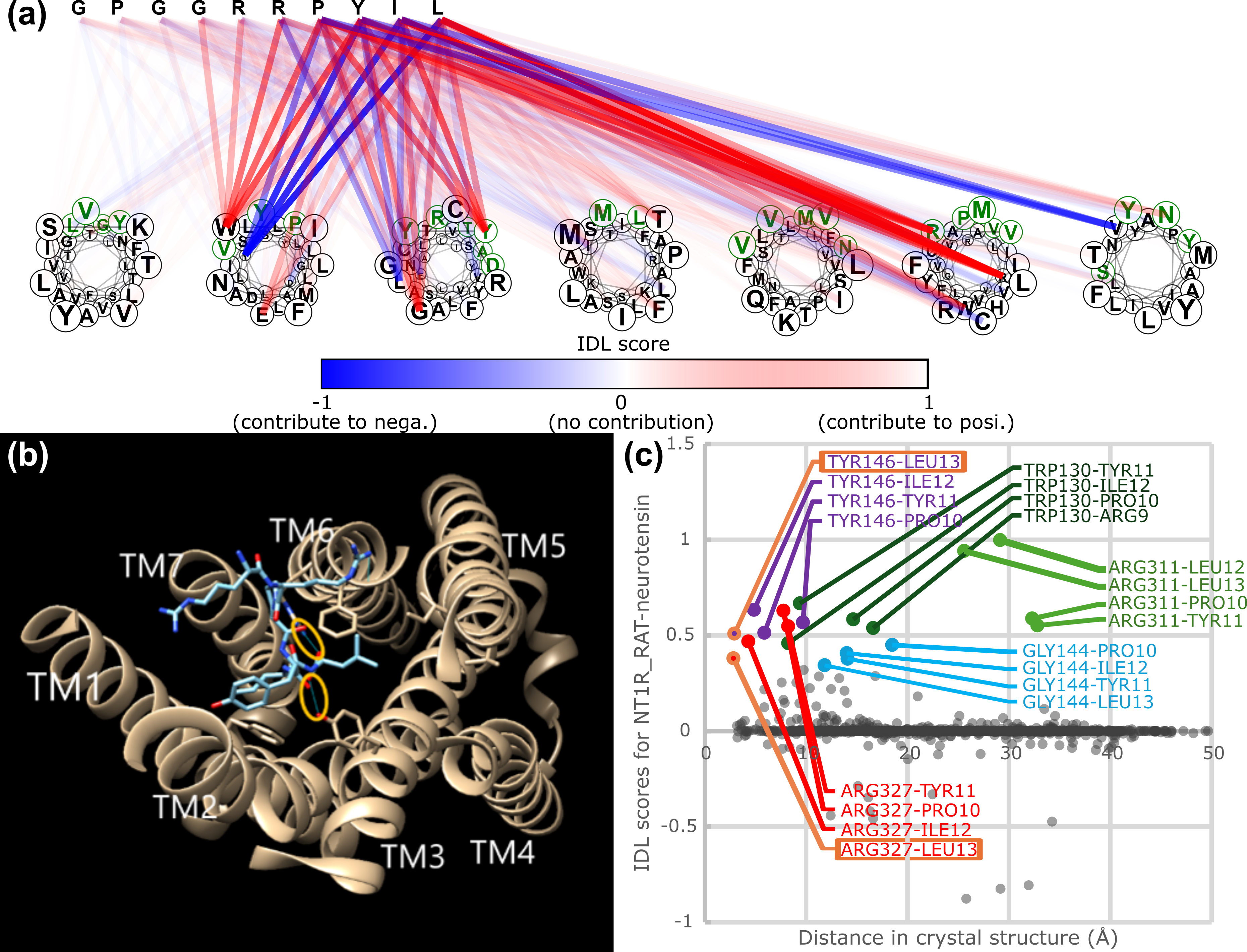
**

**Figure S3. Comparison of the IDL scores of the NTR1-neurotensin pair and the distances in the cocrystallized structure.** (A) IDL scores of the NTR1-neurotensin pair. Transmembrane residues are plotted with circles, and cavity-exposed residues are highlighted in green. Contribution scores of each peptide and GPCR residue pair are displayed in the heatmap. (B) Cocrystallized tertiary structure of NTR1 and neurotensin 1 (PDBID:4BUO). Hydrogen bonds between ligand and receptor are highlighted in orange. (C) Scatter plot of IDL scores of GPCR-peptide residue pairs and physical distances in the cocrystallized tertiary structure. Residue pairs with high IDL scores are plotted in different colors based on receptor residue position and Hydrogen bond-forming residue pairs were highlighted in orange.

**
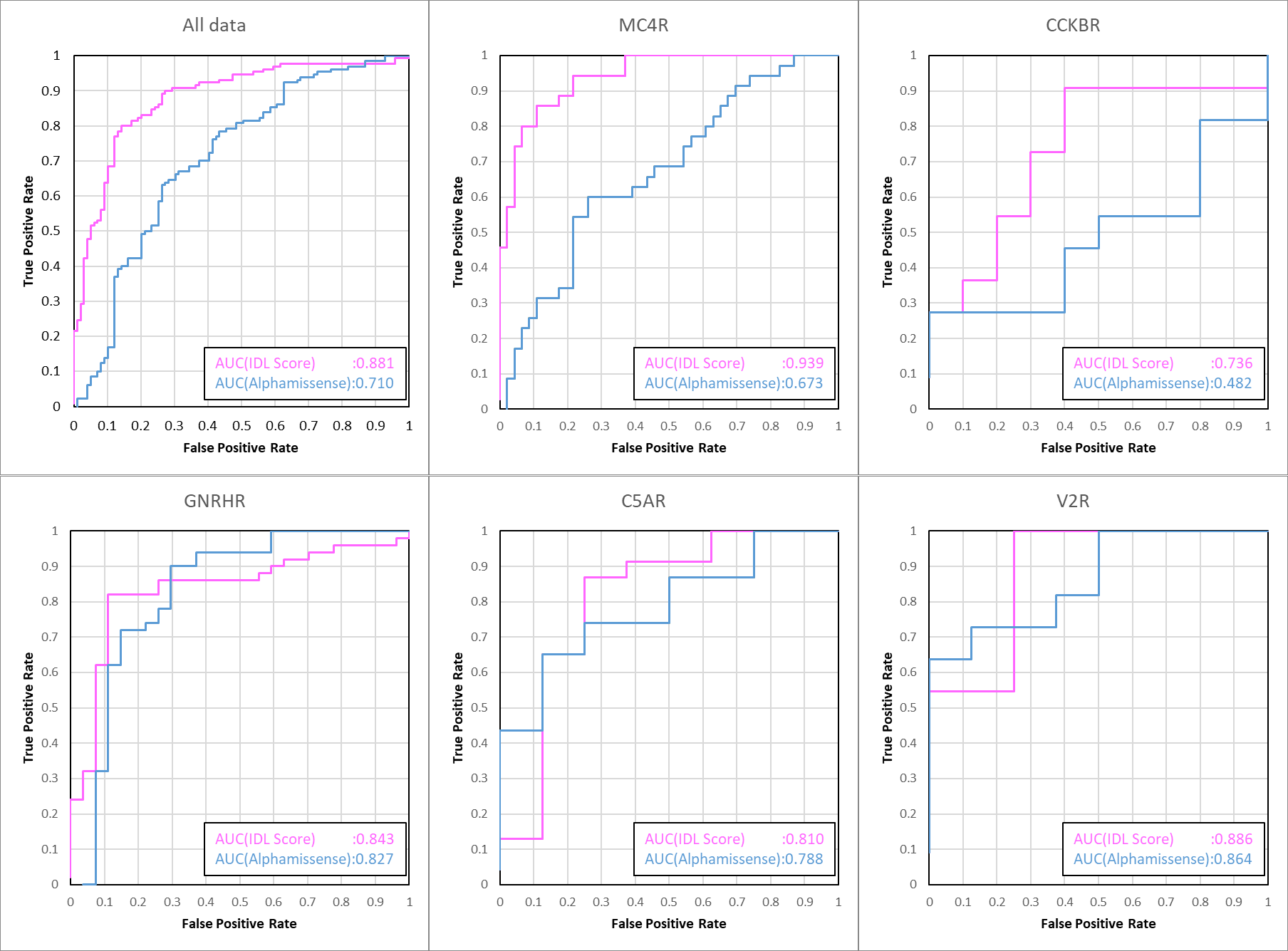
**

**Figure S4. ROC curve of IDL score- and Alphamissense-based interaction-decreasing mutation.** ROC curve for IDL score and Alphamissense were shown in pink and blue. Area under ROC curve for them were also noted in the same color.


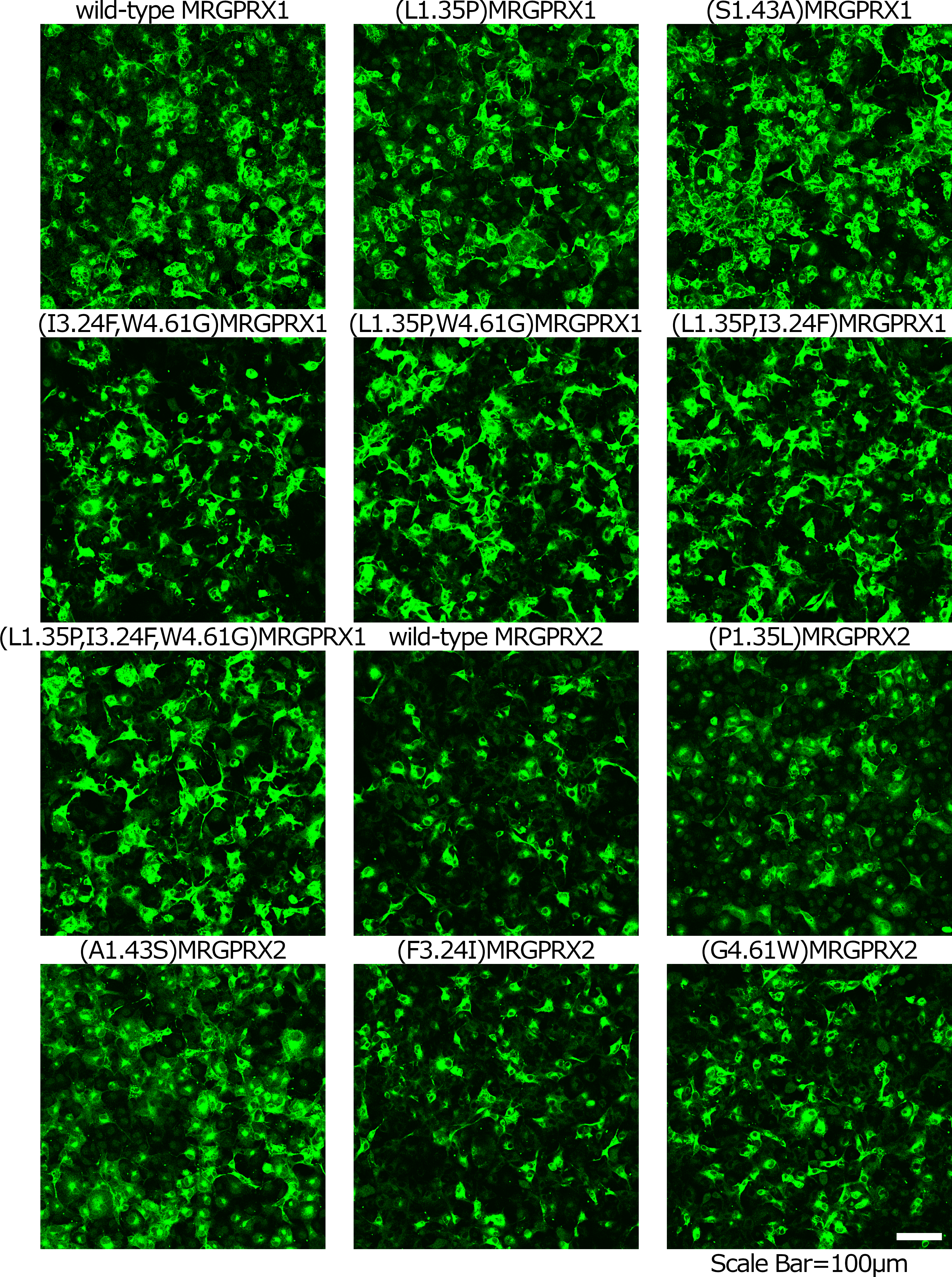


**Figure S5. Subcellular localization of MRGPRX1/2 mutants.** Immunostaining using the GNA15-specific antibody followed by treatment with Alexa 488-conjugated secondary antibody (green). These results confirmed that all of the mutated MRGPRXs are expressed to a similar degree. Scale bar, 100μm.


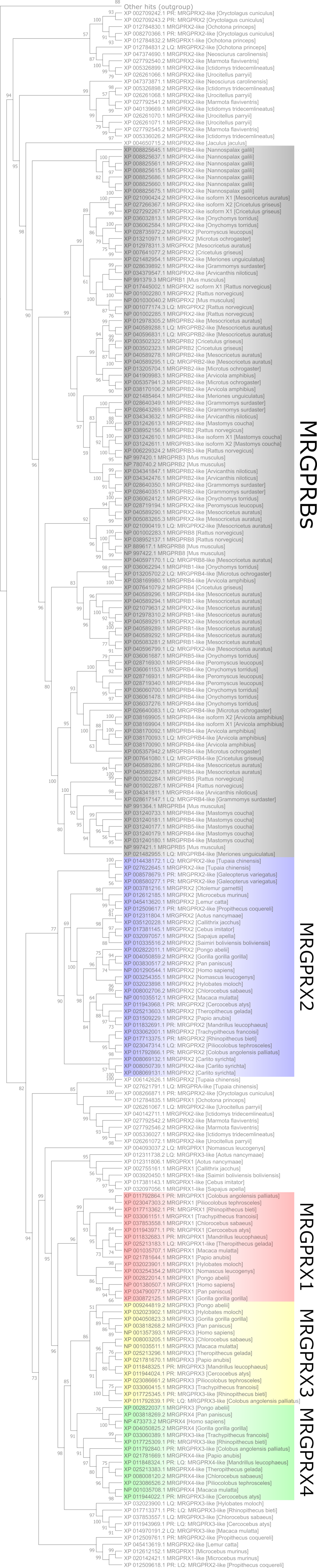


**Figure S6. Gene trees of MRGPRX1-4 and collected sequences.** The determinant residues for BAM 8-22 (L1.35) and SP (F3.24 and G4.61) are highlighted in red and blue respectively.

**Supplementary Tables**

**Table S1. Primers for the cloning and mutation of MRGPRX1/2.**

| primers for wild type MRGPRX1/2 | |
| --- | --- |
| MRGPRX2 FW | AGA CGC GGC CGC ATG GAT CCA ACC ACC CCG |
| MRGPRX2 RV | CCT TCT AGA CAC CAG ACT GCT TCT CGA CA |
| MRGPRX1 FW | GAG GAA TTC ATG GAT CCA ACC ATC TCA AC |
| MRGPRX1 RV | CCT TCT AGA CTG CTC CAA TCT GCT TCC CGA |
| primers for PDE mutants | |
| (L1.35P)-MRGPRX1 FW | CAG ACC TTG AGC CCC ACG GTG CTG |
| (L1.35P)-MRGPRX1 RV | CAG CAC CGT GGG GCT CAA GGT CTG |
| (I3.24F)-MRGPRX1 FW | TAT CCC CCA TAC CTT CTC TAA AAT CCT CTA |
| (I3.24F)-MRGPRX1 RV | TAG AGG ATT TTA GAG AAG GTA TGG GGG ATA |
| (W4.61G)-MRGPRX1 FW | CAT CCT GGA GGG GAT GTT ATG TG |
| (W4.61G)-MRGPRX1 RV | CAC ATA ACA TCC CCT CCA GGA TG |
| (S1.43A)-MRGPRX1 FW | TGC ATC GTT GCC CTT GTC GGG CTG ACA GGA AAC GCG GTT GTG CTC TG |
| (S1.43A)-MRGPRX1 RV | CGA CAA GGG CAA CGA TGC ACG TCA GCA CCG TGA GGC TCA AGG TCT GCT |
| (L1.35P,S1.43)-AMRGPRX1 FW | TGC ATC GTT GCC CTT GTC GGG CTG ACA GGA AAC GCG GTT GTG CTC TG |
| (L1.35P,S1.43)-AMRGPRX1 RV | CGA CAA GGG CAA CGA TGC ACG TCA GCA CCG TGG GGC TCA AGG TCT GCT |
| (P1.35L)-MRGPRX2 FW | GAC CCT GAT CCT GGT CTT CCT GA |
| (P1.35L)-MRGPRX2 RV | TCA GGA AGA CCA GGA TCA GGG TC |
| (F3.24I)-MRGPRX2 FW | CTC CAT CAA TAT CCC TAG CTT CTT CA |
| (F3.24I)-MRGPRX2 RV | TGA AGA AGC TAG GGA TAT TGA TGG AG |
| (G4.61W)-MRGPRX2 FW | AGC ATC TTG GAA TGG AAG TTC TGT G |
| (G4.61W)-MRGPRX2 RV | CAC AGA ACT TCC ATT CCA AGA TGC T |
| (A1.43S)-MRGPRX2 FW | TTC ATT TCC CTG GTC GGG CTG GTA GGA AAC GGG |
| (A1.43S)-MRGPRX2 RV | GAC CAG GGA AAT GAA AAG GAT CAG GAA GAC CGG |
| (P1.35L,A1.43S)-MRGPRX2 FW | TTC ATT TCC CTG GTC GGG CTG GTA GGA AAC GGG |
| (P1.35L,A1.43S)-MRGPRX2 RV | GAC CAG GGA AAT GAA AAG GAT CAG GAA GAC CAG |

**Supporting References**

1. Hayakawa, E., Guzman, C., Horiguchi, O., Kawano, C., Shiraishi, A., Mohri, K., Lin, M.-F., Nakamura, R., Nakamura, R., and Kawai, E. (2022) Mass spectrometry of short peptides reveals common features of metazoan peptidergic neurons. *Nature ecology & evolution*. **6**, 1438–1448

2. Shiraishi, A., Okuda, T., Miyasaka, N., Osugi, T., Okuno, Y., Inoue, J., and Satake, H. (2019) Repertoires of G protein-coupled receptors for *Ciona* -specific neuropeptides. *Proc. Natl. Acad. Sci. U.S.A.* **116**, 7847–7856

3. Ballesteros, J. A., and Weinstein, H. (1995) [19] Integrated methods for the construction of three-dimensional models and computational probing of structure-function relations in G protein-coupled receptors. *Methods in neurosciences*, **25**, 366–428

4. White, J. F., Noinaj, N., Shibata, Y., Love, J., Kloss, B., Xu, F., Gvozdenovic-Jeremic, J., Shah, P., Shiloach, J., and Tate, C. G. (2012) Structure of the agonist-bound neurotensin receptor. *Nature*. **490**, 508–513

5. Bumbak, F., Thomas, T., Noonan-Williams, B. J., Vaid, T. M., Yan, F., Whitehead, A. R., Bruell, S., Kocan, M., Tan, X., Johnson, M. A., Bathgate, R. A. D., Chalmers, D. K., Gooley, P. R., and Scott, D. J. (2020) Conformational Changes in Tyrosine 11 of Neurotensin Are Required to Activate the Neurotensin Receptor 1. *ACS Pharmacol. Transl. Sci.* **3**, 690–705

6. Kato, H. E., Zhang, Y., Hu, H., Suomivuori, C.-M., Kadji, F. M. N., Aoki, J., Krishna Kumar, K., Fonseca, R., Hilger, D., and Huang, W. (2019) Conformational transitions of a neurotensin receptor 1–Gi1 complex. *Nature*. **572**, 80–85

7. Lee, S., Bhattacharya, S., Tate, C. G., Grisshammer, R., and Vaidehi, N. (2015) Structural Dynamics and Thermostabilization of Neurotensin Receptor 1. *J. Phys. Chem. B*. **119**, 4917–4928

8. Yang, Y., Fong, T. M., Dickinson, C. J., Mao, C., Li, J.-Y., Tota, M. R., Mosley, R., Van Der Ploeg, L. H. T., and Gantz, I. (2000) Molecular Determinants of Ligand Binding to the Human Melanocortin-4 Receptor. *Biochemistry*. **39**, 14900–14911
